# Compassion satisfaction and fatigue: emotional responses to nursing professionals’ work and health

**DOI:** 10.1590/0034-7167-2024-0615

**Published:** 2025-12-08

**Authors:** Margani Cadore Weis Maia, Moisés Kogien, Vanessa Ferraz Leite, Victor Hugo Martins Santos, Marina Nolli Bittencourt

**Affiliations:** I Universidade Federal de Mato Grosso. Cuiabá, Mato Grosso, Brazil; II Secretaria de Estado de Saúde de Roraima, Hospital Geral de Roraima. Boa Vista, Roraima, Brazil; III Universidade do Estado de Mato Grosso. Tangará da Serra, Mato Grosso, Brazil

**Keywords:** Compassion Fatigue, Mental Health, Occupational Health, Nurse Practitioners, Hospitals, University., Desgaste por Empatía, Salud Mental, Salud Laboral, Enfermeras Practicantes, Hospitales Universitarios.

## Abstract

**Objectives:**

to analyze the association between emotional responses in health work and physical and mental health conditions with compassion satisfaction and compassion fatigue in nursing professionals from a Brazilian capital.

**Methods:**

a cross-sectional study, carried out from August 2023 to July 2024 with 95 nursing professionals from a public hospital. Professional Quality of Life, Depression, Anxiety, and Stress scales and characterization questionnaires were applied. The analysis was carried out by effect size and multiple linear regression.

**Results:**

satisfaction was associated with depression, desire to change profession, indifference to distress, and meaning of life at work. Fatigue was related to stress, alcohol use, skills to intervene in distress, and perception that working conditions impact willingness to care.

**Conclusions:**

negative emotional responses at work and mental health are associated with lower satisfaction and greater compassion fatigue in nursing professionals.

## RESUMEN

**Objetivos:**

analizar la asociación entre las respuestas emocionales en el trabajo sanitario y las condiciones de salud física y mental con la satisfacción por compasión y la fatiga por compasión en profesionales de enfermería de una capital brasileña.

**Métodos:**

estudio transversal, realizado entre agosto de 2023 y julio de 2024 con 95 profesionales de enfermería de un hospital público. Se aplicaron las escalas de *Professional Quality of Life, Depression, Anxiety, and Stress*, así como cuestionarios de caracterización. El análisis se realizó mediante el tamaño del efecto y regresión lineal múltiple.

**Resultados:**

la satisfacción se asoció con la depresión, el deseo de cambiar de profesión, la indiferencia ante el sufrimiento y el sentido de la vida en el trabajo. La fatiga se relacionó con el estrés, el consumo de alcohol, las habilidades para intervenir ante el sufrimiento y la percepción de que las condiciones laborales influyen en la disposición a cuidar.

**Conclusiones:**

las respuestas emocionales negativas en el trabajo y la salud mental se asocian con una menor satisfacción y una mayor fatiga por compasión en los profesionales de enfermería.

## INTRODUCTION

Compassion fatigue (CF) refers to the adverse emotional consequences caused by prolonged exposure to stress arising from compassionate work and is considered a major occupational hazard among nursing workers^(1-5)^. This condition can result in physical, mental, and emotional symptoms related to work experiences, which significantly affect workers’ quality of care, work performance, staff-patient relationships and quality of life^(3-7)^. CF is composed of two elements: burnout, characterized by exhaustion, frustration, anger and depression; and secondary traumatic stress (STS), marked by fear and the repercussions of exposure to trauma in the work context^(2)^.

In parallel, compassion satisfaction (CS) refers to feeling effective in one’s work, whether by helping others through care or by contributing to the work and social environment^(2,8)^. CS is considered a moderator of CF and a protective factor against occupational psychological risks^(2,9,10)^. Both are part of professional quality of life (PQoL) model, and negative implications on PQoL are expressed by a low level of CS concomitant with high levels of CF (high levels of burnout and STS)^(2)^.

The increase in care demands and the often-stressful working conditions in nursing, such as caring for patients with complex needs in environments with limited resources, combined with the difficulty in managing feelings and emotions that emerge from the care relationship, influence the decrease in CS and the occurrence of CF^(10-14)^. The impact of PQoL tends to be more severe when the precarious work environment is combined with weakened nursing professionals’ physical, psychological and social aspects, which amplifies the negative effects on their health and work performance^(5,15)^.

As a result, these workers may experience declines in physical and mental health and overall well-being, as well as negative emotional responses to work, such as apathy, disconnection, and impaired ability to exercise compassion, ultimately compromising patient care quality^(5,11,12,14,16)^.

Empathetic and compassionate nursing professionals are essential for healthcare services to provide high-quality, humane care^(11,15,17,18)^. To this end, it is necessary to identify and reduce the factors that contribute to the decline in PQoL, in order to promote CS and protect professionals from the undesirable effects of CF^(10,19)^. In this study, we hypothesized that unfavorable emotional responses in the context of health work and worse perceptions of physical and mental health among nursing professionals will be associated with lower CS scores and higher CF scores.

## OBJECTIVES

To analyze the association between emotional responses in health work and physical and mental health conditions with CS and CF in nursing professionals in a Brazilian capital.

## METHODS

### Ethical aspects

The research was approved by the Research Ethics Committee, under Certificate of Presentation for Ethical Consideration 71245823.2.0000.5541 and Opinion 6.204.287, and was conducted in accordance with the ethical guidelines established by Resolution 466/2012 of the Brazilian National Health Council (In Portuguese, *Conselho Nacional de Saúde* - CNS). All participants signed the Informed Consent Form (ICF), which ensured anonymity, confidentiality of information and the right to withdraw at any time, without prejudice or embarrassment.

### Study design, period and location

This is an analytical cross-sectional study conducted between August 2023 and July 2024, in accordance with the STrengthening the Reporting of OBservational studies in Epidemiology guidelines^(20)^. The research was conducted in a medium-sized general public university hospital located in a capital city in the Brazilian Midwest. The institution has 116 beds, including 18 intensive care beds (adult and neonatal) and more than 50 specialty clinics, offering outpatient care, hospitalizations, diagnostic and therapeutic support services, emergency care, and health surveillance. It has been part of the *Empresa Brasileira de Serviços Hospitalares* network since 2013, and has been a reference in the care of severe cases of COVID-19 in the region.

### Population and sample; inclusion and exclusion criteria

The study population consisted of all nursing professionals, nurses and nursing technicians working at the institution during the data collection period (N = 367). All of these were invited to participate, regardless of their area of activity or function, thus characterizing an unintentional sample. Nursing professionals with at least six months of practical experience were considered eligible for the study, aiming to ensure prior exposure to contexts potentially related to CS and CF, and those who responded incompletely to the research data collection instruments were excluded.

To ensure that all potential subjects for the study sample were surveyed, regular visits were made to all sectors in all institutional shifts to publicize the study and invite participation. Moreover, weekly reminders were sent to professionals’ email addresses and messaging apps during the data collection period. However, 100 professionals participated, which corresponds to a participation rate of approximately 27.4% of the target population. Of the 100 respondents, five were excluded because they did not fully meet the eligibility criteria adopted, resulting in a final sample of 95 participants.

Before conducting inferential analyses, a sample power calculation was performed for multiple linear regression analysis using the GPower version 3.1.9.7, in order to verify whether the number of professionals recruited was sufficient to guarantee a sample power of 80% and enable valid statistical inferences from the analyses performed and thus mitigate the chances of committing a type II error, i.e., failure to detect a true effect when it actually exists. The analysis demonstrated that the sample size recruited was sufficient, considering the small effect size (0.08), the significance level of 95%, the sample power of 80% and a model with up to four retained predictors.

### Study protocol

The study was disseminated over a four-month period (August to December 2023) via the institutional website and by sending participation link to the institutional email of the eligible population by the hospital’s social communication team. The link was also shared by the nursing management team in the institution’s WhatsApp^®^ groups. In addition, folders with a QR code to access the form were distributed throughout all shifts and sectors of the hospital by research team members (scientific initiation students, graduate students, and advisors).

Data collection was performed by the main researcher using Google Forms^®^. The ICF was made available and signed online by respondents who, after reading it, had to select the checkbox indicating that they were aware of the terms and agreed to participate in the study. After acceptance, participants were asked to enter an email address so that a copy of the ICF could be automatically sent to participants. The form was configured so that the email address was not recorded, and spreadsheets were organized by a researcher other than the one who performed data analysis, ensuring anonymity and confidentiality at all stages of the research. After acceptance, participants completed the instruments in an estimated average time of 20 minutes.

The study instruments included: Professional Quality of Life^(2)^: self-reported scale, adapted to Brazil^(21)^, which measures PQoL through its components (CS and CF), in 28 items and two subscales: CS, with 15 items, CF, composed of burnout symptoms, with three items, and STS, with ten items. Responses are of Likert type, between 0 (never) and 5 (always), and the final score (continuous) varies between 0 and 75 points, in which higher scores indicate a greater occurrence of the phenomenon. Cut-off points are set around the 25^th^ and 75^th^ percentiles, based on the database^(2)^. The interpretation of PQoL should take into account the scores of both components so that low CS scores (<25%), combined with moderate to high CF scores (>25%), indicate an imbalance in PQoL.

In this study, the instrument reliability was tested using McDonald’s omega, which attested to adequate internal consistency for the sample studied (CS ω = 0.831; STS ω = 0.879; and burnout ω = 0.736).

The sociodemographic variables questionnaire, constructed by the authors, was composed of questions related to sex, age, race/color, sexual orientation, length of work, unit of activity and occupation.

The questionnaire of self-reported variables of emotional responses to health work was composed of an instrument constructed by the authors for application in the context of this study, based on emotional manifestations/responses of CF (stress due to empathy, distancing, exhaustion and depersonalization, persistent excitement, loss of social identity) and CS (empathic attitude, healthy bonds, satisfaction with one’s work performance)^(2,21,22)^. It addresses the following issues: willingness to change profession and sector; perceptions of working conditions regarding willingness to care for patients; physical and emotional abilities to intervene in distress; obligation and willingness to act to alleviate patients’ distress; feelings of anger and indifference when providing care; difficulty concentrating on work activities; irritability or outbursts of anger during care work; perception of work as a source of meaning in life; and emotional distress when leaving work. The answers to the questions were dichotomous (“yes”/”no”).

The questionnaire on variables related to perception of health conditions was constructed by the authors to characterize participants’ perception of their physical and mental health conditions, including general perception of health, physical activity, presence of preexisting diseases, use of medications for insomnia or for symptoms of depression, anxiety and stress (DAS), alcohol use and number of reported sleep disorders. Responses ranged from two (“yes”/”no”) to three options (“rarely”, “frequently”, “never”). Both instruments were pre-tested by seven clinical nurses and researchers before their application, in order to ensure the comprehensibility of statements as well as the time required to complete them.

Furthermore, the Depression, Anxiety, and Stress Scale (DASS-21) was used as one of the indicators of the sample’s mental health conditions. This is a self-report instrument that assesses DAS symptoms^(23)^, adapted to Brazil^(24)^, and consists of 21 items with responses on a Likert scale, from 0 to 3 points, divided into three subscales, each with seven items. The higher the total score of each subscale, the greater the symptoms. The reliability for the three subscales was tested in this study using McDonald’s omega, which indicated adequate internal consistency (depression ω = 0.885; anxiety ω = 0.891; and stress ω = 0.911).

### Data analysis and statistics

Data were extracted from Google Forms^®^ with double checking. Variables were organized and named according to the codebook. The data were stored on a secure platform with an access password and were analyzed anonymously. The results were presented in an aggregated manner, in order to prevent individual identification of participants.

Comparative analyses between the mean CS and CF scores and dichotomous variables were performed using t-tests for independent samples, adopting a 95% confidence level and assumption of homogeneity of variance using Levene’s test. For polytomous variables, analysis of variance (ANOVA) was used, with Games-Howell post-hoc test, to identify differences between groups. Continuous variables were analyzed using Pearson’s correlation test. The analyses showed that the 95% Confidence Intervals were obtained through bootstrapping with 1,000 resamples (bias-corrected and accelerated confidence interval)^(25)^.

Due to the small sample size of this study, the interpretation of relevant variables in bivariate analyses was carried out through analyses of the effect magnitude measures (Cohen’s d for t-tests and eta squared for ANOVA), with variables with moderate or high effect magnitude being considered important, regardless of p-values.

Multiple analysis of associated factors was performed using the multiple linear regression technique. To construct the multiple model, all explanatory variables that presented a p-value <0.20 or moderate/high effect sizes in bivariate analysis were tested, and these were introduced simultaneously using the backward technique. Non-significant variables in the multiple analysis were removed one by one, until only the variables that presented a p-value <0.05 remained in the final model. Before adopting the final model, the assumptions were checked, including checking the normality of the distribution of residuals, checking for the absence of multicollinearity using the Variance Inflation Factor less than 10, and confirming the absence of autocorrelation of residuals using the Durbin-Watson test. The analyses were processed using the Statistical Package for the Social Sciences version 27.

## RESULTS

The study included the participation of 95 nursing professionals. Among the sociodemographic and work characteristics, the following stood out: women (83.2%), age over 40 years (55.8%), self-declared as black or brown (63.2%), heterosexual (89.5%) and nurses (50.5%). Concerning the scores of PQoL components, the sample presented high CS levels (average of 52.89 points) and moderate CF levels (average of 23.86 points).

In relation to self-reported variables about emotional responses to healthcare work, most participants reported a desire to change profession (57.9%) and sector (61.1%). Most stated that working conditions affected their willingness to provide care (70.5%). Furthermore, almost all stated that they felt an obligation to act to alleviate distress (95.8%), stating that they had the skills to intervene in distress (94.7%). They also confirmed having difficulty concentrating (77.9%) and irritability (71.6%) during their work activities.

Still regarding the variables of emotional responses to work, most denied having the desire to act to alleviate distress (76.8%), denied feeling angry (58.9%) and denied indifference (72.6%) during patient care activities. The denial that work gives meaning to life (67.4%) and the denial that being away from work was a cause of distress (44.2%) also predominated. Table 1 presents the details of these variables and the differences in the mean scores of the CS and CF subscales.

**Table 1:** Comparison between mean scores of compassion satisfaction and compassion fatigue and emotional responses related to health work in a sample of nursing professionals (N=95), Cuiabá, Mato Grosso, Brazil, 2024

ERW	n (%)	Satisfaction through compassion					Compassion fatigue				
		Mean (SD^*^)	t/F test†	*p* value	BCaCI95%^‡^	Size effect	Mean (SD^*^)	t/F test†	*p* value	BCaCI95%^‡^	Size effect
**Desire to change profession**											
Yes	55 (57.9%)	51.42 (5.52)	3.257	0.003	1.50; 5.45	0.68	27.47 (12.86)	-3.283	0.002	-13.50; -3.21	0.68
No	40 (42.1%)	54.92 (4.68)					18.90 (12.15)				
**Desire to change sectors**											
Yes	58 (61.1%)	52.30 (5.45)	1.356	0.155	-0.81; 3.80	0.28	26.27 (13.03)	-2.279	0.031	-12.05; -0.54	0.48
No	37 (38.9%)	53.84 (5.35)					20.08 (12.74)				
**Working conditions affect willingness to care**											
Yes	67 (70.5%)	52.37 (5.41)	1.454	0.152	-0.68; 4.22	0.33	26.64 (13.50)	-3.790^§^	0.001	-14.11; -4.97	0.75
No	28 (29.5%)	54.14 (5.38)					17.21 (9.85)				
**Skills to intervene in distress**											
Yes	90 (94.7%)	52.90 (5.52)	-0.040	0.968	-5.08; 4.88	0.02	24.45 (13.16)	-1.880	0.003	-21.29; -3.12	0.86
No	05 (5.3%)	52.80 (4.15)					13.20 (9.60)				
**Obligation to act to alleviate distress**											
Yes	91 (95.8%)	53.13 (5.15)	-2.062	0.210	-18.13; 4.85	1.05	24.43 (13.08)	-2.064	0.001	-23.27; -2.70	1.05
No	04 (4.2%)	47.50 (9.47)					10.75 (9.53)				
**Willingness to act to alleviate distress**											
Yes	22 (23.2%)	52.32 (5.57)	0.565	0.573	-1.69; 3.22	0.14	25.25 (13.75)	2.215^§^	0.030	0.28; 11.27	0.54
No	73 (76.8%)	53.07 (5.43)					19.27 (10.15)				
**Feels angry when caring**											
Yes	39 (41.1%)	51.15 (5.26)	2.688	0.003	0.93; 5.19	0.56	25.92 (13.07)	-1.273	0.183	-9.62; 2.16	0.27
No	56 (58.9%)	54.10 (5.26)					22.43 (13.21)				
**Feel indifference to distress**											
Yes	26 (27.4%)	50.42 (6.33)	2.817	0.022	0.86; 6.16	0.65	27.46 (15.12)	-1.645	0.128	-11.58; 0.61	0.38
No	69 (72.6%)	53.82 (4.79)					22.50 (12.25)				
**Difficulty concentrating during work activities**											
Yes	74 (77.9%)	55.61 (3.66)	2.685	0.004	1.47; 5.43	0.84	24.95 (13.06)	-1.530	0.137	-10.94; 1.10	0.38
No	21 (22.1%)	52.12 (5.63)					20.00 (13.28)				
**Feel irritable during work activities**											
Yes	68 (71.6%)	52.28 (5.33)	1.770	0.090	-0.60; 4.53	0.40	26.13 (12.55)	-2.749	0.006	-13.46; -2.04	0.63
No	27 (28.4%)	54.44 (5.50)					18.15 (13.31)				
**Work gives meaning to life**											
Yes	31 (32.6%)	50.70 (6.48)	2.824	0.016	0.89; 5.81	0.62	26.39 (14.12)	-1.301	0.196	-9.82; 2.43	0.29
No	64 (67.4%)	53.95 (4.55)					22.64 (12.67)				
**Taking time off work caused emotional distress**											
Yes	23 (24.2%)	52.04 (4.53)	1.025†	0.363	-	0.02	31.17 (11.75)^ab^	5.253†	0.007^||^	-	0.10
No	42 (44.2%)	52.54 (6.62)					22.28 (10.55)^a^				
Not applicable	30 (31.6%)	54.03 (4.02)					20.46 (15.65)^b^				

ERW - emotional responses to health work;

* standard deviation; †F statistic, when the ANOVA test was used; ‡95% Confidence Interval corrected and accelerated for bias; §non-homogeneous variances detected by the Levene test; ||statistically significant differences between groups identified in the Games-Howell post-hoc test (pairs of identical letters indicate different means); BCaCI - bias-corrected and accelerated confidence interval.

As for CS, analysis of the effect sizes obtained shows that feeling an obligation to act to alleviate distress (d=1.05) and stating difficulty concentrating during work activities (d=0.84) were the emotional responses with the greatest clinical or practical significance, both with effect sizes considered large. In addition to these, denying having a desire to change profession (d=0.68), denying feeling angry when caring (d=0.56), denying indifference to the distress of those who care (d=0.65) and denying that work gives meaning to life (d=0.62) also demonstrated clinical potential, presenting effect sizes of moderate magnitude.

Therefore, these professionals who feel obliged to act and have difficulty concentrating presented lower CS levels than the comparative group, which highlights the impact of these variables and their important clinical effect on reducing positive experiences of care work.

For the CF subscale, large effect sizes were found in the variables having the skills to intervene in distress (d=0.86) and feeling obligated to act to alleviate distress (d=1.05). Furthermore, willingness to change profession (d=0.68) and to act to alleviate distress (d=0.54), feeling irritable (d=0.63), believing that working conditions affect willingness to care (d=0.75), and taking time off work caused emotional distress (η2=0.10) demonstrated moderate effect sizes in relation to CF. Thus, it is evident that these variables, skills to intervene and obligation to act, have a potentially clinically important effect, intensifying the way professionals experience CF (Table 1).

Concerning the physical and mental health conditions variables presented in Table 2, it is worth noting that a large part of the sample perceived their health as good (85.3%). In addition, they denied performing physical activity (72.6%), having pre-existing diseases (50.5%) and using medications for insomnia and DAS (66.3%), as well as consuming alcohol (69.5%). However, the majority reported having more than two sleep disorders (62.1%).

**Table 2:** Comparison between mean scores of compassion satisfaction and compassion fatigue and variables of perception of physical and mental health conditions in a sample of nursing professionals (N=95), Cuiabá, Mato Grosso, Brazil, 2024

Health cond.	n (%)	Satisfaction through compassion					Compassion fatigue				
		Mean (SD^*^)	t/F test†	*p* value	BCaCI 95%^‡^	Size effect	Mean (SD^*^)	t/F test†	*p* value	BCaCI 95%^‡^	Size effect
**Health perception**											
Poor	09 (9.5%)	53.11 (5.08)	1.002†	0.371	-	0.02	31.78 (13.39)	2.493†	0.088	-	0.05
Good	81 (85.3%)	52.67 (5.47)					23.43 (12.99)				
Excellent	05 (5.2%)	56.20 (5.54)					16.60 (12.46)				
**Perform physical activity**											
Yes	26 (27.4%)	51.21 (3.32)	0.137	0.891	-1.91; 2.08	0.05	25.89 (12.43)	-0.915	0.341	-8.22; 2.84	0.21
No	69 (72.6%)	51.44 (4.51)					23.10 (13.50)				
**Pre-existing diseases**											
Yes	47 (49.5%)	52.64 (5.20)	0.453	0.652	-1.79; 2.85	0.09	26.51 (12.57)	-1.963	0.048	-10.31; -0.08	0.40
No	48 (50.5%)	53.14 (5.70)					21.27 (13.42)				
**Use of medication for insomnia or depression, anxiety or stress**											
Yes	32 (33.7%)	53.37 (5.48)	-0.611	0.545	-3.05; 1.45	0.13	26.53 (11.01)	-1.411	0.129	-8.81; 0.92	0.31
No	63 (66.3%)	52.65 (5.44)					22.50 (14.07)				
**Alcohol use**											
Yes	29 (30.5%)	52.41 (5.45)	0.569	0.562	-1.72; 2.99	0.13	28.82 (13.40)	-2.496	0.021	-12.83; -1.46	0.55
No	66 (69.5%)	53.10 (5.46)					21.70 (12.61)				
**Sleep changes**											
0	08 (8.4%)	57.37 (3.06)^ab^	3.108†	0.049^§^		0.06	14.00 (17.53) ^a^	6.789†	0.002^§^	-	0.13
1	28 (29.5%)	52.60 (5.32)^a^					19.25 (9.02)^b^				
> 2	59 (62.1%)	52.42 (5.53) ^a^					27.39 (13.09)^ab^				

Health cond. - perception of physical and mental health conditions;

* standard deviation; †F statistic, when the ANOVA test was used; ‡95% Confidence Interval corrected and accelerated for bias; §statistically significant differences between groups identified in the Games-Howell post-hoc test (pairs of identical letters indicate different means); BCaCI - bias-corrected and accelerated confidence interval.

As for the comparisons between the mean CS and CF scores and variables of perception of health conditions, the results indicated that for CF the amount of sleep disorders (η2 = 0.13) and alcohol use (d = 0.55) were the most relevant variables, demonstrating significant differences between the scores with clinical/practical potential. As for CS, all variables of perception of physical and mental health conditions presented small effect sizes in comparisons with CS levels, indicating minimal influence of these in explaining the outcome.

The correlational analysis of CS, CF and DAS scores, presented in Table 3, indicates that CS correlated negatively with CF (r= - 0.289), depression (r= -0.341), anxiety (r= -0.171) and stress (r= -0.271). The coefficients of determination indicate shared variance values of DASS-21 domains with CS (adjusted R2 = 0.083), depression (adjusted R2 = 0.116), anxiety (adjusted R2 = 0.029) and stress (adjusted R2 = 0.073) of 8%, 11%, 3% and 7%, respectively, demonstrating that the higher the CS levels, the lower the CF and DAS levels tend to be in this population.

**Table 3:** Correlational analysis between compassion satisfaction scores, compassion fatigue and domains of the Depression, Anxiety, and Stress Scale in a sample of nursing professionals (N=95), Cuiabá, Mato Grosso, Brazil, 2024

Variables	Satisfaction through compassion	Compassion fatigue
Compassion satisfaction	1	
Compassion fatigue	-0.289^^*^^	1
Depression	-0.341^^*^^	0.492^^*^^
Anxiety	-0.171	0.520^^*^^
Stress	-0.271^^*^^	0.563^^*^^

* *Significant correlation at p<0.001 level.*

The results indicate a moderate and positive correlation between CF and depression (r = 0.492 p <0.001), and a strong and positive correlation with anxiety (r = 0.520 p <0.001) and stress (r = 0.563 p <0.001). The coefficients of determination of the correlations between CF and depression (adjusted R2 = 0.242), anxiety (adjusted R2 = 0.270) and stress (adjusted R2 = 0.316) indicate shared variance of 24%, 27% and 31.7%, respectively, highlighting the influence of mental health condition on the occurrence of CF.

Multiple linear regression analysis using the backward technique demonstrated that nursing professionals with symptoms of depression, who reported a desire to change professions, who felt indifferent to patients’ distress and who believed that work gave meaning to their lives had lower levels of CS than their comparative peers. It is noteworthy that the final model adopted was statistically significant (F = 7.787, p = <0.001, adjusted R^2^ = 0.224), indicating that the set of retained variables was able to explain 22% of the outcome (Table 4).

**Table 4:** Predictive models using multiple linear regression for factors associated with satisfaction and compassion fatigue in a sample of nursing professionals, Cuiabá, Mato Grosso, Brazil, 2024

Variables	B	Standard error		β	95%BCaCI		*p* value	VIF
					Minimum	Maximum		
**Satisfaction through compassion**								
Intercept	56.988	0.92			55.196	58.781	< 0.001	
Depression	-0.120	0.051	-0.227		-0.221	-0.020	0.019	1.103
Wants to change profession (yes)	-2.249	1.045	-0.205		-4.325	-0.174	0.034	1.101
Feels indifferent to distress (yes)	-2.549	1.120	-0.210		-4.773	-0.128	0.025	1.032
Work gives meaning to your life (yes)	-2.260	1.073	-0.196		-4.391	-0.020	0.038	1.047
**Compassion fatigue**								
Intercept	-2.189	4.942			-12.006	7.629	0.659	
Stress	0.563	0.091	0.510		0.382	0.744	<0.001	1.092
Alcohol use	6.183	2.263	0.217		1.686	10.680	0.008	1.008
Skills for intervening in distress	11.393	4.674	0.194		2.106	20.679	0.017	1.011
Working conditions affect willingness to care	5.195	2.378	0.180		0.471	9.919	0.032	1.090

*insertion method - backward; CS model (adjusted R2: 0.224; F: 7.787 (p < 0.001); Durbin-Watson - 1.747); CF model (adjusted R2: 0.413; F: 17.516 (p < 0.001); Durbin-Watson - 2.005); VIF - Variance Inflation Factor; BCaCI - bias-corrected and accelerated confidence interval.*

For CF, the same regression model demonstrated that nurses and nursing technicians with evidence of stress, who use alcohol, who declare having skills to intervene in distress and who feel that working conditions affect their willingness to care presented higher levels of CF than their comparative peers. The final model assumed was statistically significant (F = 17.516, p = < 0.00, adjusted R^2^ = 0.413), indicating that the set of retained variables was able to explain 41% of the outcome.

## DISCUSSION

This study identified that the lowest CS scores presented by nursing professionals were related to symptoms of depression, turnover intention, indifference to patients’ distress and identification of the meaning of life at work. Higher levels of CF were related to symptoms of stress, alcohol use, declared abilities to intervene in others’ distress and perception that working conditions impact willingness to care.

The literature indicates that the imbalance in PQoL has repercussions on the work performance and mental health of nursing professionals affected, who are more likely to experience depression and negative impacts on their work, such as a greater occurrence of adverse events, low quality of patient care and higher rates of turnover intention^(3,26-28)^. However, when satisfied with their work performance, they tend to have a lower risk of mental distress, since high levels of CS alleviate depressive symptoms and are strongly correlated with better levels of psychological well-being and a lower intention to leave nursing^(8,9,18,26,29)^.

The results of this study showed that CS was negatively correlated with CF, suggesting that nursing professionals with lower CS are more prone to burnout and STS. This relationship helps to explain the findings of the linear regression, which associated lower CS scores with the desire to change profession and indifference to patients’ distress. The desire to change profession reflects a negative emotional response to the work context and the desire to withdraw from the triggering place or situation^(30,31)^, consistent with avoidant behavior caused by traumatic experiences, associated with the manifestation of STS^(1,2)^. Meanwhile, indifference to patients denotes depersonalization, disconnection and insensitivity to the work environment, associated with the manifestation of burnout^(2,9,10,31)^. Although STS may, in some cases, motivate professionals to remain in nursing due to the empathic bond and the desire to support patients’ recovery, burnout is associated with work overload, and is one of the main factors that contribute to the intention to leave nursing^(18,29)^.

These behaviors of staying away from stress and not acting toward care are ways of coping with compassion stress (a precursor to CF), which tends to be alleviated by detaching from exposure to distress^(1,10,18)^. To deal with this, professionals distance themselves emotionally from their patients, but meaningful interactions for the therapeutic bond are lost^(10,11,24)^.

In contrast, some studies indicate that nursing professionals who report that work gives meaning to their lives have high levels of CS, as they are able to establish healthy empathic bonds and feel professionally fulfilled, which promotes compensation for negative experiences and greater tolerance to conflicts and frustrations that occur in their work practice^(2,8,10,31,32)^.

However, in this study, this variable (work gives meaning to life) was associated with lower CS scores. This can be explained by high levels of stress, which can lead to a loss of meaning in life at work and a drop in CS^(4,32)^. Thus, the positive feelings generated in the context of patient care may not be able to adequately alleviate burnout, which would impair the ability to act compassionately^(14,29)^.

The other variables retained in the regression model, such as symptoms of depression, desire to leave nursing and indifference, may contribute to these workers presenting lower levels of CS, as these make them less engaged and less resilient in dealing with the demanding and emotionally challenging aspects of their work^(18,27,31)^. These findings also suggest that such symptoms (depression, desire to leave nursing and indifference) among professionals with lower levels of CS reflect the presence of emotional exhaustion, which may indicate a risk of CF, highlighting the interrelationship and complexity among the PQoL domains^(2,5,7,24)^.

CF arises from the experience of prolonged exposure to a type of stress related to care work^(1,2)^, which was confirmed by the results of the linear regression of this study, which reinforced the relationship between stress and CF. Studies emphasize the positive correlation found between both, with the main consequences being a decline in well-being, loss of productivity, absenteeism due to illness, dissatisfaction and premature departure from work, contributing to the shortage of nurses^(7,12,32,33)^.

The data also indicated that the occurrence of CF was associated with professionals who reported having the skills to intervene in distress, which indicates that they have compassion to act towards patients. However, for this reason, they are also particularly vulnerable to CF, as they may be directly and deeply involved in providing multidimensional care, having a greater risk of exposure to traumatic experiences and developing a sense of extreme responsibility towards patients^(2,4,8,11,15,17)^.

In line with this, research indicates that the severity of traumatic experiences to which professionals are exposed has been associated with negative coping responses, such as the use of alcohol, in order to alleviate emotional or physical stress, which can be exacerbated by high levels of work-related stress and harmful working conditions^(10,34)^. Therefore, early recognition of STS symptoms is essential to protect nurses from psychological trauma^(4)^ and risk behaviors.

Furthermore, it was observed that the highest CF scores were associated with professionals who reported that working conditions affect their willingness to care. Compassion, being a deep awareness of others’ distress, together with the desire to alleviate it, allows nursing professionals to act therapeutically by meeting patients’ care needs, resulting in a sense of fulfillment^(1,8,17)^. However, when they feel unable to fulfill their care responsibilities due to a healthcare environment with limited resources, they end up experiencing moral distress that favors CF^(15,16)^, since poor working conditions lead to negative emotional responses, such as high levels of stress, a decrease in satisfaction with teamwork, in quality of care and in patient safety^(12)^.

Thus, the consequences of CF cannot be minimized, which makes it important to develop healthy work environments, monitor work stress and mental distress among nursing professionals, and promote PQoL strategies^(4,5,10,12,34)^. Among these, those that combine psychological, behavioral and organizational components have proven to be more effective, due to the expanded approach capable of reducing CF while strengthening CS^(11,35)^.

Possible interventions for CF should include psychological support to provide emotional release, reduce the effects of trauma and other negative emotional symptoms, as well as behavioral interventions, such as emotional regulation and stress management techniques, aimed at alleviating STS and reducing the risk of using negative coping strategies, such as alcohol consumption. Organizational actions should promote peer support, balanced distribution of work demands and improvements in the work environment, which will contribute to reducing burnout, exhaustion and professional evasion^(10,11,35)^.

These strategies tend to mitigate CF by controlling negative emotions and strengthening emotional balance, but also favor CS by promoting a healthy work environment, job satisfaction and, consequently, better quality care^(35)^. Furthermore, it is suggested that future studies examine the other strategies that nursing professionals use to manage PQoL.

### Study limitations

This study has some limitations, such as the use of a cross-sectional design, which restricts the inference of causality and temporality between variables, and the use of self-administered questionnaires, which may introduce self-report bias due to influences such as cognitive processes and social desirability. Nevertheless, this strategy was chosen for its flexibility, allowing participants to respond at the most appropriate time for their work routine and without the direct mediation of the researcher.

Furthermore, the participant selection method, which included all nursing professionals, without stratification, may have limited the generation of more specific insights into the demands of each care and professional context.

The extended data collection period, which occurred due to the difficulty of accessing professionals at the workplace and unforeseen circumstances, affected voluntary participation. Low participation may be related to the context of work pressures and emotional exhaustion, which may have compromised the representativeness of the findings, constituting a study limitation. Future research could adopt more active recruitment strategies or integrate data collection into specific institutional moments, such as team meetings or training sessions, to increase the reach and participation rate.

Another aspect to be considered concerns the way in which CF was analyzed, since although it is composed of burnout and STS dimensions, it was analyzed in the present study as a unified construct, representing the combined experience of emotional exhaustion resulting from exposure to distress in care work. However, this approach may have prevented the identification of specific factors associated with each dimension. It is recommended that future studies conduct separate analyses to deepen this understanding and guide more targeted interventions.

### Contributions to nursing

The study contributes to the knowledge of the factors that influence nursing professionals’ PQoL and emotional responses. Based on the findings of this study, the factors associated with CS and CF can be monitored and strategies focused on the identified risk and protective factors can be developed. Actions such as psychological support programs, maintenance of healthy work environments, and care interventions for stress management are also recommended.

## CONCLUSIONS

This study showed that emotional responses at work and mental health conditions are associated with satisfaction and CF in nursing professionals. Lower levels of CS were observed among those with symptoms of depression, desire to leave nursing, indifference to patients’ distress, and lower perception of meaning in work. Higher levels of CF were associated with symptoms of stress, alcohol use, perception of poor working conditions, and feeling unprepared to deal with distress. These findings reinforce the importance of institutional strategies aimed at promoting mental health and improving nursing teams’ working conditions.

## Data Availability

The research data are available within the article.

## References

[1] Figley CR. (1995). In Compassion fatigue: Coping with secondary traumatic stress disorder in those who treat the traumatized.

[2] Stamm BH. (2010). The concise ProQOL manual.

[3] Ma H, Huang SQ, We B, Zhong Y. (2022). Compassion fatigue, burnout, compassion satisfaction and depression among emergency department physicians and nurses: a cross-sectional study. BMJ Open.

[4] Koþtu N, Ýnci FH, Arslan S. (2024). Compassion fatigue and the meaning in life as predictors of secondary traumatic stress in nurses during the COVID-19 pandemic. Int J Nurs Pract.

[5] Chen X, Li J, Arber A, Qiao C, Wu J, Sun C (2024). The impact of the nursing work environment on compassion fatigue: the mediating role of general self-efficacy. Int Nurs Rev.

[6] Xie W, Chen L, Feng F, Okoli CTC, Tang P, Zeng L (2021). The prevalence of compassion satisfaction and compassion fatigue among nurses: a systematic review and meta-analysis. Int J Nurs Stud.

[7] Borges EMN, Fonseca CINS, Baptista PCP, Queirós CML, Baldonedo-Mosteiro M, Mosteiro-Diaz MP. (2019). Compassion fatigue among nurses working on an adult emergency and urgent care unit. Rev Latino-Am Enfermagem.

[8] Sacco TL, Copel LC. (2018). Compassion satisfaction: a concept analysis in nursing. Nurs Forum.

[9] Jo M, Na H, Jung YE. (2020). Mediation effects of compassion satisfaction and compassion fatigue in the relationships between resilience and anxiety or depression among hospice volunteers. J Hosp Palliat Nurs.

[10] Yeºil A, Polat ª. (2023). Investigation of psychological factors related to compassion fatigue, burnout, and compassion satisfaction among nurses. BMC Nursing.

[11] Sinclair S, Raffin-Bouchal S, Venturato L, Mijovic-Kondejewski J, Smith-MacDonald L. (2017). Compassion fatigue: a meta-narrative review of the healthcare literature. Int J Nurs Stud.

[12] AbuAlRub R, Sabei SDA, Al-Rawajfah O, Labrague LJ, Burney IA. (2023). Direct and moderating effects of work environment and structural empowerment on job stress and job satisfaction among nurses in Oman. Sultan Qaboos Univ Med J.

[13] Garnett A, Hui L, Oleynikov C, Boamah S. (2023). Compassion fatigue in healthcare providers: a scoping review. BMC Health Serv Res.

[14] Cardoso AA, Tomotani DYV, Mucci S. (2024). Compassion fatigue and coping strategies before death. Rev Bioét.

[15] Mottaghi S, Poursheikhali H, Shameli L. (2020). Empathy, compassion fatigue, guilt and secondary traumatic stress in nurses. Nurs Ethics.

[16] Ledoux K. (2015). Understanding compassion fatigue: understanding compassion. J Adv Nurs.

[17] Liu X, Li C, Yan X, Shi B. (2022). Psychological capital has a positive correlation with humanistic care ability among nurses. Front Psychol.

[18] Wei H, Cao Y, Carroll Q, Wei A, Richardson S, Nwokocha T (2024). Nursing work engagement, professional quality of life, and intent to leave: a structural equation modeling pathway analysis. J Nurs Res.

[19] Chan WCH, Tin AF, Yu TK. (2022). Professional quality of life, depression, and meaning in life among helping professionals: the moderating role of self-competence in death work. Death Studies.

[20] Von Elm EV, Altman DG, Egger M, Pocock SJ, Gøtzsche PC, Vandenbroucke JP. (2007). Strengthening the reporting of observational studies in epidemiology (STROBE) statement: guidelines for reporting observational studies. BMJ.

[21] Lago K, Codo W. (2013). Compassion fatigue: evidence of internal consistency and factorial validity in ProQol-BR. Estud Psicol (Natal).

[22] Lago K, Codo W. (2010). Fadiga por Compaixão: o sofrimento dos profissionais de saúde.

[23] Lovibond SH, Lovibond PF. (1995). Manual for the depression, anxiety and stress scales.

[24] Vignola RCB, Tucci AM. (2014). Adaptation and validation of the depression, anxiety and stress scale (DASS) to Brazilian Portuguese. J Affect Disord.

[25] Haukoos JS, Lewis RJ. (2005). Advanced Statistics: Bootstrapping Confidence Intervals for Statistics with “Difficult” Distributions. Acad Emerg Med.

[26] Pang Y, Dan H, Jung H, Bae N, Kim O. (2020). Depressive symptoms, professional quality of life and turnover intention in Korean nurses. Int Nurs Rev.

[27] Tanaka K, Ikeuchi S, Teranishi K, Oe M, Morikawa Y, Konya C. (2020). Temperament and professional quality of life among Japanese nurses. Nurs Open.

[28] Hamaideh S, Abu Khait A, Al-Modallal H, Masa’deh R, Hamdan-Mansour A, AlBashtawy M. (2024). Professional quality of life, job satisfaction, and intention to leave among psychiatric nurses: a cross-sectional study. Nurs Rep.

[29] Yu H, Gui L. (2022). Compassion fatigue, burnout and compassion satisfaction among emergency nurses: a path analysis. J Adv Nurs.

[30] Cao X, Chen L. (2021). Relationships between resilience, empathy, compassion fatigue, work engagement and turnover intention in haemodialysis nurses: a cross-sectional study. J Nurs Manag.

[31] Zhan Y, Liu Y, Chen Y, Liu H, Zhang W, Yan R (2022). The prevalence and influencing factors for compassion fatigue among nurses in Fangcang shelter hospitals: a cross-sectional study. Int J Nurs Pract.

[32] Aslan H, Erci B, Pekince H. (2022). Relationship Between Compassion Fatigue in Nurses, and Work-Related Stress and the Meaning of Life. J Relig Health.

[33] Galanis P, Moisoglou I, Katsiroumpa A, Mastrogianni M. (2024). Association between workplace bullying, job stress, and professional quality of life in nurses: a systematic review and meta-analysis. Healthcare.

[34] Jarrad R, Hammad S, Shawashi T, Mahmoud N. (2018). Compassion fatigue and substance use among nurses. Ann Gen Psychiatry.

[35] Zhang H, Xia Z, Yu S, Shi H, Meng Y, Dator WL. (2025). Interventions for compassion fatigue, burnout, and secondary traumatic stress in nurses: a systematic review and network meta-analysis. Nurs Health Sci.

