# Supplementary material for: Compassion satisfaction and fatigue: emotional responses to nursing professionals’ work and health
Source: Rev Bras Enferm. 2025 Dec 8;78(6):e20240615. doi: 10.1590/0034-7167-2024-0615 (PMC12707534; doi:10.1590/0034-7167-2024-0615)
Supplement: Translation

# Translation: Satisfação e fadiga de compaixão: respostas emocionais no trabalho e saúde de profissionais de enfermagem

**DOI:** 10.1590/0034-7167-2024-0615pt

**Authors:** Margani Cadore Weis Maia, Moisés Kogien, Vanessa Ferraz Leite, Victor Hugo Martins Santos, Marina Nolli Bittencourt

**Affiliations:** I Universidade Federal de Mato Grosso. Cuiabá, Mato Grosso, Brasil; II Secretaria de Estado de Saúde de Roraima, Hospital Geral de Roraima. Boa Vista, Roraima, Brasil; III Universidade do Estado de Mato Grosso. Tangará da Serra, Mato Grosso, Brasil

**Keywords:** Fadiga de Compaixão, Saúde Mental, Saúde Ocupacional, Profissionais de Enfermagem, Hospitais Universitários.

## Abstract

**Objetivos:**

analisar a associação entre respostas emocionais no trabalho em saúde e condições de saúde física e mental com satisfação por compaixão e fadiga de compaixão em profissionais de enfermagem de uma capital brasileira.

**Métodos:**

estudo transversal, realizado de agosto de 2023 a julho de 2024 com 95 profissionais de enfermagem de um hospital público. Aplicaram-se as escalas *Professional Quality of Life, Depression, Anxiety, and Stress* e questionários de caracterização. A análise ocorreu por magnitude de efeito e regressão linear múltipla.

**Resultados:**

a satisfação associou-se à depressão, desejo de trocar de profissão, indiferença ao sofrimento e sentido de vida no trabalho. A fadiga se relacionou ao estresse, uso de álcool, habilidades para intervir no sofrimento e percepção de que as condições de trabalho impactam a disposição para cuidar.

**Conclusões:**

respostas emocionais negativas no trabalho e saúde mental estão associadas à menor satisfação e maior fadiga de compaixão em profissionais de enfermagem.

## INTRODUÇÃO

A fadiga de compaixão (FC) refere-se às consequências emocionais adversas causadas pela exposição prolongada ao estresse oriundo do trabalho compassivo, sendo considerada um risco ocupacional importante entre trabalhadores de enfermagem^(1-5)^. Essa condição pode resultar em sintomas físicos, mentais e emocionais relacionados às vivências do trabalho, que afetam significativamente a qualidade do atendimento, o desempenho laboral, as relações equipe-paciente e a qualidade de vida dos trabalhadores^(3-7)^. A FC é composta por dois elementos: o *burnout*, caracterizado por exaustão, frustração, raiva e depressão; e o estresse traumático secundário (ETS), marcado pelo medo e pelas repercussões da exposição a traumas no contexto de trabalho^(2)^.

Em paralelo, a satisfação por compaixão (SC) refere-se ao sentir-se eficaz no seu trabalho, seja ao ajudar os outros por meio do cuidado ou ao contribuir para o ambiente laboral e social^(2,8)^. A SC é considerada moderadora da FC e fator de proteção contra riscos psicológicos ocupacionais^(2,9,10)^. Ambos integram o modelo de qualidade de vida profissional (QVP), e implicações negativas na QVP são expressas por um baixo nível de SC concomitante a níveis elevados de FC (altos níveis de *burnout* e ETS)^(2)^.

O aumento das demandas assistenciais e as condições de trabalho frequentemente estressantes em enfermagem, como o atendimento a pacientes com necessidades complexas em ambientes de recursos limitados, aliados à dificuldade de manejo de sentimentos e emoções que emergem da relação de cuidado, influenciam a diminuição da SC e ocorrência da FC^(10-14)^. O impacto da QVP tende a ser mais grave quando o ambiente de trabalho precário se combina a aspectos físicos, psicológicos e sociais debilitados dos profissionais de enfermagem, o que amplia os efeitos negativos sobre sua saúde e desempenho laboral^(5,15)^.

Como resultado, esses trabalhadores podem manifestar declínio da saúde física e mental, e do bem-estar geral, além de respostas emocionais negativas em relação ao trabalho, como apatia, desconexão e obstrução da capacidade de exercer a compaixão, comprometendo, em última análise, a qualidade do atendimento ao paciente^(5,11,12,14,16)^.

Profissionais de enfermagem empáticos e compassivos são essenciais para que os serviços de saúde forneçam atendimento humanizado e de alta qualidade^(11,15,17,18)^. Para tal, torna-se necessário identificar e reduzir os fatores que contribuem para o declínio da QVP, a fim de promover a SC e proteger os profissionais dos efeitos indesejados da FC^(10,19)^. Neste estudo, parte-se da hipótese de que respostas emocionais desfavoráveis no contexto do trabalho em saúde e piores percepções de saúde física e mental entre profissionais de enfermagem estarão associadas a menores escores de SC e maiores de FC.

## OBJETIVOS

Analisar a associação entre as respostas emocionais no trabalho em saúde e as condições de saúde física e mental com a SC e FC em profissionais de enfermagem de uma capital brasileira.

## MÉTODOS

### Aspectos éticos

A pesquisa foi aprovada pelo Comitê de Ética em Pesquisa, sob Certificado de Apresentação para Apreciação Ética nº 71245823.2.0000.5541 e Parecer nº 6.204.287, sendo conduzida em conformidade com as diretrizes éticas estabelecidas pela Resolução nº 466/2012 do Conselho Nacional de Saúde (CNS). Todos os participantes assinaram o Termo de Consentimento Livre e Esclarecido (TCLE), que assegurava o anonimato, a confidencialidade das informações e o direito de desistência a qualquer momento, sem prejuízos ou constrangimentos.

### Desenho, período e local do estudo

Trata-se de estudo transversal analítico, conduzido entre agosto de 2023 e julho de 2024, conforme as diretrizes do *STrengthening the Reporting of OBservational studies in Epidemiology* ^(20)^. A pesquisa foi realizada em um hospital universitário público geral de médio porte localizado em uma capital do centro-oeste brasileiro. A instituição conta com 116 leitos, incluindo 18 de terapia intensiva (adulto e neonatal) e mais de 50 consultórios de especialidades, oferecendo atendimentos ambulatoriais, internações, serviços de apoio diagnóstico e terapêutico, urgência, e vigilância em saúde. Integra a rede da Empresa Brasileira de Serviços Hospitalares desde 2013, e foi referência no atendimento a casos graves de COVID-19 na região.

### População e amostra; critérios de inclusão e exclusão

A população do estudo foi composta por todos os profissionais de enfermagem, enfermeiros e técnicos em enfermagem atuantes na instituição durante o período de coleta de dados (N = 367). Todos esses foram convidados a participar, independentemente da área de atuação ou função exercida, caracterizando-se, assim, uma amostra não intencional. Foram considerados elegíveis para o estudo os profissionais de enfermagem com no mínimo seis meses de atuação prática, visando assegurar exposição prévia a contextos potencialmente relacionados à SC e FC, sendo excluídos os que responderam incompletamente aos instrumentos de coleta de dados da pesquisa.

Para garantir a sondagem de todos os potenciais sujeitos para composição da amostra de estudo, foram realizadas visitas regulares a todos os setores em todos os turnos institucionais para divulgação do estudo e convite à participação. Além disso, lembretes semanais foram enviados aos endereços de e-mail e aplicativo de mensagem dos profissionais durante o período de coleta de dados. Contudo, obteve-se a adesão de 100 profissionais, o que corresponde a uma taxa de adesão de aproximadamente 27,4% da população-alvo. Dos 100 respondentes, cinco foram excluídos por não atenderem plenamente aos critérios de elegibilidade adotados, resultando em uma amostra final de 95 participantes.

Antes da condução das análises inferenciais, realizou-se cálculo de poder amostral para análise de regressão linear múltipla, por meio do *software* G*Power*, versão 3.1.9.7, a fim de verificar se o quantitativo de profissionais recrutados era suficiente para garantir poder amostral de 80% e possibilitar inferências estatísticas válidas a partir das análises realizadas e, assim, mitigar chances de cometer erro tipo II, ou seja, a falha em detectar um efeito verdadeiro quando ele realmente existe. A análise demonstrou que o tamanho amostral recrutado era suficiente, considerando o tamanho de efeito pequeno (0,08), o nível de significância de 95%, o poder amostral de 80% e um modelo com até quatro preditores retidos.

### Protocolo do estudo

A divulgação do estudo ocorreu durante quatro meses (agosto a dezembro de 2023), por meio do site institucional e do envio, pela equipe da comunicação social do hospital, do *link* de participação ao e-mail institucional da população elegível. Houve, ainda, o compartilhamento do *link*, pela gerência de enfermagem, nos grupos de *WhatsApp* ^®^ da instituição. Além disso, *folders* com *QR code* de acesso ao formulário foram distribuídos, em todos os turnos e setores do hospital, por membros da equipe de pesquisa (alunos de iniciação científica, de pós-graduação e orientadora).

A coleta de dados foi realizada, pela pesquisadora principal, por meio do *Google Forms* ^®^. O TCLE foi disponibilizado e assinado de forma *online* pelos respondentes que, após a leitura, deveriam selecionar a caixa de diálogo, indicando ciência ao termo e concordância em participar do estudo. Após o aceite, foi solicitada a inserção de um endereço de e-mail permitindo o envio automático, ao participante, de uma cópia do TCLE. O formulário foi configurado para não registrar o endereço eletrônico, e as planilhas foram organizadas por um pesquisador distinto daquele que realizou a análise dos dados, garantindo o anonimato e a confidencialidade em todas as etapas da pesquisa. Após o aceite, os participantes responderam aos instrumentos em tempo médio estimado de 20 minutos

Os instrumentos do estudo compreenderam: *Professional Quality of Life* ^(2)^: escala autorrelatada, adaptada ao Brasil^(21)^, que afere a QVP por meio de seus componentes (SC e FC), em 28 itens e duas subescalas: SC, com 15 itens, FC, composta por sintomas de *burnout*, com três itens, e ETS, com dez itens. As respostas são do tipo Likert, entre 0 (nunca) e 5 (sempre), e a pontuação final (contínua) varia entre 0 e 75 pontos, na qual maiores escores indicam maior ocorrência do fenômeno. Os pontos de cortes são fixados em torno dos percentis 25 e 75, com base no banco de dados^(2)^. A interpretação da QVP deve levar em conta a pontuação de ambos os componentes, de modo que escores baixos de SC (<25%), combinados a escores moderados a altos de FC (>25%), indicam um desequilíbrio na QVP.

Neste estudo, a confiabilidade do instrumento foi testada por meio do ômega de McDonald, que atestou adequada consistência interna para a amostra estudada (SC ω = 0,831; ETS ω = 0,879; e *burnout* ω = 0,736).

O questionário de variáveis sociodemográficas, construído pelos autores, foi composto por questões relacionadas ao sexo, idade, raça/cor, orientação sexual, tempo de trabalho, unidade de atuação e ocupação.

O questionário de variáveis autorrelatadas das respostas emocionais do trabalho em saúde foi composto por instrumento construído pelos autores para aplicação no contexto deste estudo, embasado nas manifestações/respostas emocionais da FC (estresse por empatia, distanciamento, exaustão e despersonalização, excitação persistente, perda de identidade social) e da SC (atitude empática, vínculos salutares, contentamento com sua atuação laboral)^(2,21,22)^. Aborda as seguintes questões: vontade de trocar de profissão e de setor; percepções das condições de trabalho sobre a disposição para cuidar dos pacientes; habilidades físicas e emocionais para intervir no sofrimento; obrigação e vontade de agir para aliviar o sofrimento dos pacientes; sentimentos de raiva e indiferença ao cuidar; dificuldades de concentração nas atividades laborais; irritabilidade ou acesso de raiva durante o trabalho de cuidado; percepção do trabalho como fonte de sentido para a vida; e sofrimento emocional ao afastar-se do trabalho. As respostas às perguntas foram dicotômicas (“sim”/“não”).

O questionário de variáveis de percepção de condições de saúde foi construído pelos autores para caracterizar a percepção dos participantes sobre suas condições de saúde física e mental, incluindo a percepção geral da saúde, prática de atividade física, presença de doenças preexistentes, uso de medicamentos para insônia ou para sintomas de depressão, ansiedade e estresse (DAS), uso de álcool e número de alterações de sono relatadas. As respostas variaram entre duas (“sim”/”não”) e três opções (“raramente”, “frequentemente”, “nunca”). Ambos os instrumentos foram pré-testados por sete enfermeiros clínicos e pesquisadores antes da sua aplicação, a fim de assegurar a compressibilidade das afirmativas, bem como o tempo de preenchimento.

Além disso, a *Depression, Anxiety, and Stress Scale* (DASS-21) foi utilizada como um dos indicadores das condições de saúde mental da amostra. Trata-se de instrumento de autorrelato que avalia sintomas de DAS^(23)^, adaptado ao Brasil^(24)^, composto por 21 itens com respostas em escala tipo Likert, de 0 a 3 pontos, divididos em três subescalas, cada uma com sete itens. Quanto maior o escore total de cada subescala, maiores são os sintomas. A confiabilidade para as três subescalas foi testada neste estudo por meio do ômega de McDonald, que indicou adequada consistência interna (depressão ω = 0,885; ansiedade ω = 0,891; e estresse ω = 0,911).

### Análise de dados e estatística

Os dados foram extraídos do *Google Forms* ^®^ com dupla conferência. As variáveis foram organizadas e nominadas conforme *codebook*. Os dados foram armazenados em plataforma segura com senha de acesso, sendo analisados de forma anonimizada. Os resultados foram apresentados de maneira agregada, de modo a impedir a identificação individual dos participantes.

Análises comparativas entre os escores médios de SC e FC e as variáveis dicotômicas foram realizadas por meio de testes *t* para amostras independentes, adotando-se nível de confiança de 95% e pressuposto de homogeneidade de variância por meio do teste de Levene. Para as variáveis politômicas, empregou-se análise de variância (ANOVA), com teste *post-hoc* de Games-Howell, para identificar diferenças entre grupos. Variáveis contínuas foram analisadas pelo teste de correlação de Pearson. As análises mostraram que os Intervalos de Confiança de 95% foram obtidos por meio de *bootstrapping* com 1.000 reamostragens (*bias-corrected and accelerated confidence interval*)^(25)^.

Devido ao pequeno tamanho amostral deste estudo, a interpretação das variáveis relevantes nas análises bivariadas se deu por meio das análises das medidas de magnitude de efeito (d de Cohen para os testes t e o eta ao quadrado para ANOVA), sendo consideradas importantes as variáveis com magnitude de efeito moderada ou alta, independentemente dos valores de p.

Análise múltipla de fatores associados se deu por meio de técnica de regressão linear múltipla. Para construção do modelo múltiplo, foram testadas todas as variáveis explicativas que apresentaram valor de p<0,20 ou tamanhos de efeitos moderados/altos na análise bivariada, tendo essas sido introduzidas simultaneamente por meio da técnica *backward*. As variáveis não significativas na análise múltipla foram removidas uma a uma, até restarem retidas no modelo final apenas as variáveis que apresentaram valor de p<0,05. Antes de assumir o modelo final, realizaram-se verificações dos pressupostos, incluindo a conferência da normalidade da distribuição dos resíduos, a verificação da ausência de multicolinearidade por meio do *Variance Inflation Factor* menor que 10 e a confirmação de não ocorrência de autocorrelação de resíduos por meio do teste de Durbin-Watson. As análises foram processadas no *software Statistical Package for the Social Science*, versão 27.

## RESULTADOS

O estudo contou com a participação de 95 profissionais de enfermagem. Entre as características sociodemográficas e laborais, destacaram-se predominância de mulheres (83,2%), idade acima de 40 anos (55,8%), autodeclarados como pretos ou pardos (63,2%), heterossexuais (89,5%) e enfermeiros (50,5%). Em relação aos escores dos componentes da QVP, a amostra apresentou níveis elevados de SC (média de 52,89 pontos) e moderados de FC (média de 23,86 pontos).

Em relação às variáveis autorrelatadas acerca das respostas emocionais do trabalho em saúde, a maioria dos participantes referiu desejo de trocar de profissão (57,9%) e de setor (61,1%). Grande parte afirmou que as condições de trabalho afetaram a sua disposição para cuidar (70,5%). Além disso, quase a totalidade afirmou sentir a obrigação de agir para aliviar o sofrimento (95,8%), afirmando possuir habilidades para intervir no sofrimento (94,7%). Também confirmaram sentir dificuldade de concentração (77,9%) e irritabilidade (71,6%) durante suas atividades laborais.

Ainda em relação às variáveis de respostas emocionais do trabalho, grande parte negou ter vontade de agir para aliviar o sofrimento (76,8%), negou sentir raiva (58,9%) e negou indiferença (72,6%) durante as atividades de cuidado ao paciente. Predominou, ainda, a negação de que o trabalho dá um sentido à vida (67,4%), e a negação de que se afastar do trabalho foi motivo de sofrimento (44,2%). O detalhamento dessas variáveis e as diferenças das pontuações médias das subescalas de SC e FC são apresentadas na Tabela 1.

**Tabela 1:** Comparação entre escores médios de satisfação por compaixão e fadiga de compaixão e respostas emocionais relacionados ao trabalho em saúde em uma amostra de profissionais de enfermagem (N=95), Cuiabá, Mato Grosso, Brasil, 2024

RET	n (%)	Satisfação por compaixão					Fadiga de compaixão				
		Média (DP^*^)	Teste t/F†	Valor de *p*	BCaCI95%^‡^	Tamanho efeito	Média (DP^*^)	Teste t/F†	Valor de *p*	BCaCI95%^‡^	Tamanho efeito
**Vontade de trocar de profissão**											
Sim	55 (57,9%)	51,42 (5,52)	3,257	0,003	1,50; 5,45	0,68	27,47 (12,86)	-3,283	0,002	-13,50; -3,21	0,68
Não	40 (42,1%)	54,92 (4,68)					18,90 (12,15)				
**Vontade de trocar de setor**											
Sim	58 (61,1%)	52,30 (5,45)	1,356	0,155	-0,81; 3,80	0,28	26,27 (13,03)	-2,279	0,031	-12,05; -0,54	0,48
Não	37 (38,9%)	53,84 (5,35)					20,08 (12,74)				
**Condições de trabalho afetam a disposição para cuida**r											
Sim	67 (70,5%)	52,37 (5,41)	1,454	0,152	-0,68; 4,22	0,33	26,64 (13,50)	-3,790^§^	0,001	-14,11; -4,97	0,75
Não	28 (29,5%)	54,14 (5,38)					17,21 (9,85)				
**Habilidades para intervir no sofrimento**											
Sim	90 (94,7%)	52,90 (5,52)	-0,040	0,968	-5,08; 4,88	0,02	24,45 (13,16)	-1,880	0,003	-21,29; -3,12	0,86
Não	05 (5,3%)	52,80 (4,15)					13,20 (9,60)				
**Obrigação de agir para aliviar o sofrimento**											
Sim	91 (95,8%)	53,13 (5,15)	-2,062	0,210	-18,13; 4,85	1,05	24,43 (13,08)	-2,064	0,001	-23,27; -2,70	1,05
Não	04 (4,2%)	47,50 (9,47)					10,75 (9,53)				
**Vontade de agir para aliviar o sofrimento**											
Sim	22 (23,2%)	52,32 (5,57)	0,565	0,573	-1,69; 3,22	0,14	25,25 (13,75)	2,215^§^	0,030	0,28; 11,27	0,54
Não	73 (76,8%)	53,07 (5,43)					19,27 (10,15)				
**Sente raiva ao cuida**r											
Sim	39 (41,1%)	51,15 (5,26)	2,688	0,003	0,93; 5,19	0,56	25,92 (13,07)	-1,273	0,183	-9,62; 2,16	0,27
Não	56 (58,9%)	54,10 (5,26)					22,43 (13,21)				
**Sente indiferença ao sofrimento**											
Sim	26 (27,4%)	50,42 (6,33)	2,817	0,022	0,86; 6,16	0,65	27,46 (15,12)	-1,645	0,128	-11,58; 0,61	0,38
Não	69 (72,6%)	53,82 (4,79)					22,50 (12,25)				
**Dificuldade de concentração durante suas atividades laborais**											
Sim	74 (77,9%)	55,61 (3,66)	2,685	0,004	1,47; 5,43	0,84	24,95 (13,06)	-1,530	0,137	-10,94; 1,10	0,38
Não	21 (22,1%)	52,12 (5,63)					20,00 (13,28)				
**Sente irritabilidade durante as atividades laborais**											
Sim	68 (71,6%)	52,28 (5,33)	1,770	0,090	-0,60; 4,53	0,40	26,13 (12,55)	-2,749	0,006	-13,46; -2,04	0,63
Não	27 (28,4%)	54,44 (5,50)					18,15 (13,31)				
**O trabalho dá um sentido à vida**											
Sim	31 (32,6%)	50,70 (6,48)	2,824	0,016	0,89; 5,81	0,62	26,39 (14,12)	-1,301	0,196	-9,82; 2,43	0,29
Não	64 (67,4%)	53,95 (4,55)					22,64 (12,67)				
**Afastar-se do trabalho provocou sofrimento emocional**											
Sim	23 (24,2%)	52,04 (4,53)	1,025†	0,363	-	0,02	31,17 (11,75)^ab^	5,253†	0,007^||^	-	0,10
Não	42 (44,2%)	52,54 (6,62)					22,28 (10,55)^a^				
Não se aplica	30 (31,6%)	54,03 (4,02)					20,46 (15,65)^b^				

RET - respostas emocionais ao trabalho em saúde;

* desvio padrão; †estatística F, quando se empregou o teste ANOVA;

‡ Intervalo de Confiança de 95% corrigido e acelerado por viés;

§ variâncias não homogêneas detectadas pelo teste de Levene;

|| diferenças estatisticamente significativas entre grupos identificadas no teste post-hoc de Games-Howell (pares de letras iguais indicam médias diferentes); BCaCI - bias-corrected and accelerated confidence interval.

Sobre a SC, análise dos tamanhos de efeito obtidos demonstra que sentir obrigação de agir para aliviar o sofrimento (d=1,05) e afirmar dificuldade de concentração durante suas atividades laborais (d=0,84) foram as respostas emocionais com maior significância clínica ou prática, ambas com tamanhos de efeito considerados grandes. Além destas, negar ter vontade de trocar de profissão (d=0,68), negar sentir raiva ao cuidar (d= 0,56), negar indiferença ao sofrimento de quem cuida (d=0,65) e negar que o trabalho dá um sentido à vida (d=0,62) demonstraram, também, potencial clínico, apresentando tamanhos de efeito de magnitude moderada.

Logo, esses profissionais que sentem obrigação de agir e dificuldade de concentração apresentaram níveis de SC inferiores aos do grupo comparativo, o que evidencia o impacto dessas variáveis e seu efeito clínico importante na redução das experiências positivas do trabalho de cuidado.

Para a subescala FC, tamanhos de efeito grande foram encontradas nas variáveis possuir habilidades para intervir no sofrimento (d= 0,86) e sentir obrigação de agir para aliviar o sofrimento (d= 1,05). Ainda, vontade de trocar de profissão (d=0,68) e de agir para aliviar o sofrimento (d=0,54), sentir irritabilidade (d=0,63), acreditar que as condições de trabalho afetam a disposição para cuidar (d=0,75), e afastar-se do trabalho provocou sofrimento emocional (η2=0,10) demonstraram tamanho de efeito moderado em relação à FC. Dessa forma, evidencia-se que essas variáveis, habilidades para intervir e obrigação de agir, têm um potencial efeito clinicamente importante, intensificando a maneira como os profissionais vivenciam a FC (Tabela 1).

Sobre as variáveis de condições de saúde física e mental apresentadas na Tabela 2, destaca-se que grande parte da amostra percebeu sua saúde como boa (85,3%). Além disso, negaram realizar atividade física (72,6%), doenças pré-existentes (50,5%) e uso de medicamentos para insônia e DAS (66,3%), bem como o consumo de álcool (69,5%). No entanto, a maioria relatou possuir mais de duas alterações de sono (62,1%).

**Tabela 2:** Comparação entre escores médios de satisfação por compaixão e fadiga de compaixão e variáveis de percepção de condições de saúde física e mental em uma amostra de profissionais de enfermagem (N=95), Cuiabá, Mato Grosso, Brasil, 2024

Cond. de saúde	n (%)	Satisfação por compaixão					Fadiga de compaixão				
		Média (DP^*^)	Teste t/F†	Valor de *p*	BCaCI 95%^‡^	Tamanho efeito	Média (DP^*^)	Teste t/F†	Valor de *p*	BCaCI 95%^‡^	Tamanho efeito
**Percepção de saúde**											
Ruins	09 (9,5%)	53,11 (5,08)	1,002†	0,371	-	0,02	31,78 (13,39)	2,493†	0,088	-	0,05
Boas	81 (85,3%)	52,67 (5,47)					23,43 (12,99)				
Excelentes	05 (5,2%)	56,20 (5,54)					16,60 (12,46)				
**Realiza atividade física**											
Sim	26 (27,4%)	51,21 (3,32)	0,137	0,891	-1,91; 2,08	0,05	25,89 (12,43)	-0,915	0,341	-8,22; 2,84	0,21
Não	69 (72,6%)	51,44 (4,51)					23,10 (13,50)				
**Doenças preexistente**s											
Sim	47 (49,5%)	52,64 (5,20)	0,453	0,652	-1,79; 2,85	0,09	26,51 (12,57)	-1,963	0,048	-10,31; -0,08	0,40
Não	48 (50,5%)	53,14 (5,70)					21,27 (13,42)				
**Uso de medicamento para insônia ou para depressão ansiedade ou estresse**											
Sim	32 (33,7%)	53,37 (5,48)	-0,611	0,545	-3,05; 1,45	0,13	26,53 (11,01)	-1,411	0,129	-8,81; 0,92	0,31
Não	63 (66,3%)	52,65 (5,44)					22,50 (14,07)				
**Uso de álcool**											
Sim	29 (30,5%)	52,41 (5,45)	0,569	0,562	-1,72; 2,99	0,13	28,82 (13,40)	-2,496	0,021	-12,83; -1,46	0,55
Não	66 (69,5%)	53,10 (5,46)					21,70 (12,61)				
**Alterações de sono**											
0	08 (8,4%)	57,37 (3,06)^ab^	3,108†	0,049^§^		0,06	14,00 (17,53) ^a^	6,789†	0,002^§^	-	0,13
1	28 (29,5%)	52,60 (5,32)^a^					19,25 (9,02)^b^				
> 2	59 (62,1%)	52,42 (5,53) ^a^					27,39 (13,09)^ab^				

Cond. de saúde - percepção de condições de saúde física e mental;

* desvio padrão; †estatística F, quando se empregou o teste ANOVA;

‡ Intervalo de Confiança de 95% corrigido e acelerado por viés;

§ diferenças estatisticamente significativas entre grupos identificadas no teste post-hoc de Games-Howell (pares de letras iguais indicam médias diferentes); BCaCI - bias-corrected and accelerated confidence interval.

Em relação às comparações entre os escores médios de SC e FC e variáveis de percepção de condições de saúde, os resultados indicaram que, para FC, a quantidade de alterações de sono (η2 =0,13) e uso de álcool (d=0,55) foram as variáveis mais relevantes, demonstrando diferenças significativas entre os escores com potencial clínico/prático. Quanto à SC, todas as variáveis de percepção das condições de saúde física e mental apresentaram tamanhos de efeito pequenos nas comparações com os níveis de SC, indicando influência mínima dessas na explicação do desfecho.

A análise correlacional dos escores de SC, FC e DAS, apresentado na Tabela 3, indica que a SC se correlacionou de maneira negativa com FC (r= - 0,289), depressão (r= -0,341), ansiedade (r= -0,171) e estresse (r= -0,271). Os coeficientes de determinação indicam valores de variância compartilhada dos domínios da DASS-21 com SC (R^2^ ajustado =0,083), depressão (R^2^ ajustado = 0,116), ansiedade (R^2^ ajustado = 0,029) e estresse (R^2^ ajustado = 0,073) de 8%, 11%, 3% e 7%, respectivamente, demonstrando que quanto maiores forem os níveis de SC, menores tendem a ser os níveis de FC e de DAS nessa população.

**Tabela 3:** Análise correlacional entre os escores de satisfação por compaixão, fadiga de compaixão e domínios da *Depression, Anxiety, and Stress Scale* em uma amostra de profissionais de enfermagem (N=95), Cuiabá, Mato Grosso, Brasil, 2024

Variáveis	Satisfação por compaixão	Fadiga de compaixão
Satisfação por compaixão	1	
Fadiga de compaixão	-0,289^^*^^	1
Depressão	-0,341^^*^^	0,492^^*^^
Ansiedade	-0,171	0,520^^*^^
Estresse	-0,271^^*^^	0,563^^*^^

* *Correlação significativa a nível p<0,001.*

Os resultados indicam haver uma correlação moderada e positiva da FC com depressão (r= 0,492 p <0,001), e forte e positiva com ansiedade (r= 0,520 p < 0,001) e estresse (r= 0,563 p< 0,001). Os coeficientes de determinação das correlações entre FC e depressão (R^2^ ajustado = 0,242), ansiedade (R^2^ ajustado = 0,270) e estresse (R^2^ ajustado = 0,316) indicam variância compartilhada de 24%, 27% e 31,7%, respectivamente, destacando a influência da condição de saúde mental na ocorrência da FC.

A análise de regressão linear múltipla por técnica *backward* demonstrou que os profissionais de enfermagem com sintomas de depressão, que relatam vontade de trocar de profissão, que se sentem indiferentes ao sofrimento dos pacientes e que acham que o trabalho dá um sentido a sua vida tiveram menores níveis de SC do que seus pares comparativos. Destaca-se que o modelo final assumido foi estatisticamente significativo (F =7,787, p =<0,001, R^2^ ajustado =0,224), indicando que o conjunto de variáveis retidas conseguiu explicar 22% do desfecho (Tabela 4).

**Tabela 4:** Modelos preditivos por regressão linear múltipla para fatores associados à satisfação e fadiga de compaixão em uma amostra de profissionais de enfermagem, Cuiabá, Mato Grosso, Brasil, 2024

Variáveis	B	Erro padrão		β	BCaIC95%		Valor de *p*	VIF
					Mínimo	Máximo		
**Satisfação por compaixão**								
Intercepto	56,988	0,92			55,196	58,781	< 0,001	
Depressão	-0,120	0,051	-0,227		-0,221	-0,020	0,019	1,103
Vontade de trocar de profissão (sim)	-2,249	1,045	-0,205		-4,325	-0,174	0,034	1,101
Sente-se indiferente ao sofrimento (sim)	-2,549	1,120	-0,210		-4,773	-0,128	0,025	1,032
O trabalho dá sentido à sua vida (sim)	-2,260	1,073	-0,196		-4,391	-0,020	0,038	1,047
**Fadiga de compaixão**								
Intercepto	-2,189	4,942			-12,006	7,629	0,659	
Estresse	0,563	0,091	0,510		0,382	0,744	<0,001	1,092
Uso de álcool	6,183	2,263	0,217		1,686	10,680	0,008	1,008
Habilidades para intervir no sofrimento	11,393	4,674	0,194		2,106	20,679	0,017	1,011
Condições de trabalho afetam disposição de cuidar	5,195	2,378	0,180		0,471	9,919	0,032	1,090

*método de inserção - backward; modelo SC (R2 ajustado: 0,224; F: 7,787 (p <0,001); Durbin-Watson - 1,747); modelo FC (R2 ajustado: 0,413; F: 17,516 (p < 0,001); Durbin-Watson - 2,005); VIF - Variance Inflation Factor; BCaCI - bias-corrected and accelerated confidence interval.*

Para FC, o mesmo modelo de regressão demonstrou que enfermeiros e técnicos de enfermagem com evidências de estresse, que fazem uso de álcool, que declaram possuir habilidades para intervir no sofrimento e que sentem que as condições de trabalho afetam sua disposição de cuidar apresentaram maiores níveis de FC do que seus pares comparativos. O modelo final assumido foi estatisticamente significativo (F =17,516, p = < 0,00, R^2^ ajustado= 0,413,) indicando que o conjunto de variáveis retidas conseguiu explicar 41% do desfecho.

## DISCUSSÃO

Este estudo identificou que os menores escores de SC apresentados pelos profissionais de enfermagem estavam relacionados a sintomas de depressão, intenção de rotatividade, indiferença ao sofrimento dos pacientes e identificação do sentido da vida no trabalho. Maiores níveis de FC foram relacionados a sintomas de estresse, uso de álcool, habilidades declaradas para intervir no sofrimento alheio e percepção de que as condições de trabalho impactam a disposição para cuidar.

A literatura aponta que o desequilíbrio na QVP repercute na atuação laboral e saúde mental dos profissionais de enfermagem afetados, estando esses mais sujeitos a vivenciar depressão e impactos negativos no trabalho, como maior ocorrência de eventos adversos, baixa qualidade no atendimento ao paciente e maiores taxas de intenção de rotatividade^(3,26-28)^. Todavia, quando satisfeitos com sua atuação laboral, tendem a possuir menor risco de sofrimento mental, uma vez que altos níveis de SC atenuam sintomas depressivos e correlacionam-se fortemente a melhores índices de bem-estar psicológico e menor intenção de deixar a profissão^(8,9,18,26,29)^.

Os resultados deste estudo apontaram que a SC se correlacionou negativamente à FC, sugerindo que profissionais de enfermagem com menor SC estão mais propensos ao *burnout* e ETS. Essa relação ajuda a explicar os achados da regressão linear, que associaram menores escores em SC à vontade de trocar de profissão e à indiferença diante do sofrimento dos pacientes. O desejo de mudança de profissão reflete uma resposta emocional negativa ao contexto do trabalho e ao desejo de se retirar do local ou situação de gatilho^(30,31)^, compatível com o comportamento evitativo originado por experiências traumáticas, associado à manifestação do ETS^(1,2)^. Enquanto isso, a indiferença aos pacientes denota despersonalização, desconexão e insensibilidade ao ambiente de trabalho, associada à manifestação do *burnout* ^(2,9,10,31)^. Embora o ETS possa, em alguns casos, motivar os profissionais a permanecerem na profissão devido ao vínculo empático e ao desejo de apoiar a recuperação dos pacientes, o *burnout* está associado à sobrecarga de trabalho, e é um dos principais fatores que contribuem para a intenção de deixar a enfermagem^(18,29)^.

Esses comportamentos de manter distância do estresse e não agir em direção ao cuidado são formas de enfrentamento do estresse da compaixão (precursor da FC), que tende a ser aliviado por meio do desligamento da exposição ao sofrimento^(1,10,18)^. Para lidar com isso, os profissionais se distanciam emocionalmente de seus pacientes, porém as interações significativas para o vínculo terapêutico são perdidas^(10,11,24)^.

Em contrapartida, alguns estudos apontam que os profissionais de enfermagem que relatam que o trabalho dá um sentido à sua vida apresentaram níveis elevados de SC, pois conseguem estabelecer vínculos empáticos salutares e sentir-se realizados profissionalmente, o que promove compensação às experiências negativas, maior tolerância aos conflitos e frustrações que ocorrem em sua prática laboral^(2,8,10,31,32)^.

Neste estudo, no entanto, essa variável (o trabalho dá sentido à vida) foi associada aos escores mais baixos de SC. Isso pode ser explicado pelos altos níveis de estresse, que podem levar à perda do sentido de vida no trabalho e à queda da SC^(4,32)^. Dessa forma, os sentimentos positivos gerados no contexto do cuidado ao paciente podem não ser capazes de aliviar adequadamente o esgotamento, o que prejudicaria a capacidade de agir de forma compassiva^(14,29)^.

As outras variáveis retidas no modelo de regressão, como sintomas de depressão vontade de sair da profissão e indiferença, podem colaborar para que esses trabalhadores apresentem níveis mais baixos de SC, já que estas os tornam menos engajados e menos resilientes para lidar com os aspectos exigentes e emocionalmente desafiadores de seu trabalho^(18,27,31)^. Esses achados sugerem ainda que tais sintomas (depressão, desejo de deixar a profissão e indiferença) entre os profissionais com menores níveis de SC refletem a presença de exaustão emocional, o que pode indicar risco de FC, evidenciando a inter-relação e a complexidade entre os domínios da QVP^(2,5,7,24)^.

A FC advém da experiência de exposição prolongada a um tipo de estresse relacionado ao trabalho de cuidado^(1,2)^, o que foi confirmado pelos resultados da regressão linear deste estudo, que reforçou a relação entre estresse e FC. Estudos enfatizam a correlação positiva encontrada entre ambos, tendo como principais consequências o declínio do bem-estar, perda de produtividade, absenteísmo por doença, insatisfação e saída prematura no trabalho, contribuindo para a escassez de enfermeiros^(7,12,32,33)^.

Os dados ainda apontaram que a ocorrência de FC foi associada a profissionais que relataram ter habilidades para intervir no sofrimento, o que denota que esses dispõem de compaixão para agir em direção aos pacientes. No entanto, por esse motivo, também são particularmente vulneráveis à FC, pois podem se envolver de forma direta e profunda no fornecimento de cuidados multidimensionais, tendo maior risco de exposição a experiências traumáticas e ao desenvolvimento de um senso de responsabilidade extrema em relação aos pacientes^(2,4,8,11,15,17)^.

Em consonância, pesquisas apontam que a gravidade das experiências traumáticas às quais os profissionais são expostos tem se associado a respostas negativas de enfrentamento, como o uso de álcool, no intuito de aliviar o estresse emocional ou físico, que podem ser potencializadas por níveis elevados de estresse laboral e deletérias condições de trabalho^(10,34)^. Assim, o reconhecimento precoce dos sintomas de ETS é essencial para proteger os enfermeiros de traumas psicológicos^(4)^ e de comportamentos de risco.

Ainda, foi observado que os maiores escores de FC foram associados a profissionais que relataram que as condições de trabalho afetam a disposição para cuidar. A compaixão, sendo uma profunda consciência do sofrimento do outro, juntamente com o desejo de aliviá-lo, permite que os profissionais de enfermagem atuem terapeuticamente atendendo às necessidades de cuidado dos pacientes, resultando em um senso de realização^(1,8,17)^. No entanto, quando estes se percebem incapazes de cumprir sua responsabilidade de cuidado devido a um ambiente de saúde com recursos limitados, acabam vivenciando um sofrimento moral que favorece a FC^(15,16)^, uma vez que as condições precárias de trabalho levam a respostas emocionais negativas, como níveis elevados de estresse, queda na satisfação no trabalho em equipe, na qualidade do atendimento e na segurança do paciente^(12)^.

Assim, as consequências da FC não podem ser minimizadas, o que torna importante desenvolver ambientes saudáveis de trabalho, monitoramento do estresse laboral e do sofrimento mental dos profissionais de enfermagem, e estratégias de promoção da QVP^(4,5,10,12,34)^. Entre essas, aquelas que combinam componentes psicológicos, comportamentais e organizacionais têm se mostrado mais eficazes, pela abordagem ampliada capaz de reduzir a FC enquanto fortalecem a SC^(11,35)^.

Possíveis intervenções para a FC devem incluir apoio psicológico, a fim de propiciar liberação emocional, redução dos efeitos de traumas e dos demais sintomas emocionais negativos, bem como intervenções comportamentais, como técnicas de regulação emocional e gerenciamento do estresse, visando aliviar o ETS e diminuir o risco de uso de estratégias de enfrentamento negativas, como o consumo de álcool. Ações organizacionais devem promover o apoio entre os pares, distribuição equilibrada das demandas laborais e melhorias no ambiente de trabalho, que contribuirão para a redução do esgotamento, do *burnout* e da evasão profissional^(10,11,35)^.

Essas estratégias tendem a mitigar a FC, ao controlar emoções negativas e fortalecer o equilíbrio emocional, mas também favorecer a SC, devido à promoção de ambiente de trabalho saudável, satisfação profissional e, consequentemente, cuidados de melhor qualidade^(35)^. Ademais, sugere-se que estudos futuros examinem as demais estratégias que os profissionais de enfermagem utilizam para o gerenciamento da QVP.

### Limitações do estudo

Este estudo apresenta algumas limitações, como o uso de desenho transversal, o que restringe a inferência de causalidade e temporalidade entre as variáveis e a utilização de questionários autoaplicáveis, que podem introduzir viés de autorrelato, devido a influências como processos cognitivos e desejabilidade social. Ainda assim, essa estratégia foi escolhida por sua flexibilidade, permitindo que os participantes respondessem no momento mais adequado à sua rotina laboral e sem a mediação direta do pesquisador.

Além disso, o método de seleção dos participantes, que incluiu todos os profissionais de enfermagem, sem estratificação, pode ter limitado a geração de *insights* mais específicos sobre as demandas de cada contexto assistencial e profissional.

O período estendido de coleta de dados, que ocorreu devido à dificuldade de acesso aos profissionais nos locais de trabalho e de imprevistos, afetou a adesão voluntária. A baixa participação pode estar relacionada ao contexto de pressões laborais e esgotamento emocional, o que pode ter comprometido a representatividade dos achados, configurando uma limitação do estudo. Pesquisas futuras poderiam adotar estratégias mais ativas de recrutamento ou integrar a coleta a momentos institucionais específicos, como reuniões de equipe ou capacitações, para ampliar o alcance e a taxa de participação.

Outro aspecto a ser considerado diz respeito à forma de análise da FC, pois embora esta seja composta pelas dimensões de *burnout* e ETS, foi analisada, no presente estudo, enquanto construto unificado, representando a experiência combinada de exaustão emocional resultante da exposição ao sofrimento no trabalho de cuidado. No entanto, essa abordagem pode ter impedido a identificação de fatores específicos associados a cada dimensão. Recomenda-se que estudos futuros realizem análises separadas para aprofundar essa compreensão e orientar intervenções mais direcionadas.

### Contribuições para a área da enfermagem

O estudo contribui para o conhecimento dos fatores que influenciam a QVP e as respostas emocionais dos profissionais de enfermagem. A partir dos achados deste estudo, podem ser realizados o monitoramento dos fatores associados à SC e FC e a construção de estratégias focadas nos fatores de risco e protetivos identificados. Também são recomendadas ações como programas de apoio psicológico, manutenção de ambientes de trabalho salutares e intervenções de cuidado para a gestão do estresse.

## CONCLUSÕES

Este estudo evidenciou que respostas emocionais no trabalho e condições de saúde mental estão associadas à satisfação e FC em profissionais de enfermagem. Menores níveis de SC foram observados entre aqueles com sintomas de depressão, desejo de deixar a profissão, indiferença ao sofrimento dos pacientes e menor percepção de sentido no trabalho. Já os maiores níveis de fadiga de compaixão se associaram a sintomas de estresse, uso de álcool, percepção de más condições laborais e sensação de possuir preparo para lidar com o sofrimento. Esses achados reforçam a importância de estratégias institucionais voltadas à promoção da saúde mental e à melhoria das condições de trabalho da equipe de enfermagem.

## Data Availability

Os dados da pesquisa estão disponíveis no corpo do artigo.
